# Pregnancy-Associated Melanoma: A Molecular Reappraisal of the Hormonal Hypothesis, Illustrated by a Postpartum-Persistent Melanoma In Situ

**DOI:** 10.3390/diagnostics16152485

**Published:** 2026-08-06

**Authors:** Laura Maghiar, Andrada Iftode, Andreea-Adriana Neamțu, Teodor-Andrei Maghiar, Raul Chioibas, Diana Haj-Ali, Cristina Dumitrescu, Ciprian-Nicușor Solomon, Valentin-Cristian Iovin, Ovidiu Tica, Anca Huniadi, Cristina-Adriana Dehelean, Ilarie Brihan

**Affiliations:** 1 Department of Psycho-Neurosciences and Rehabilitation, Faculty of Medicine and Pharmacy, University of Oradea, Universității Str., No. 1, 410087 Oradea, Romania; laura.maghiar@uoradea.ro (L.M.); ibrihan@uoradea.ro (I.B.); 2 Department of Dermatovenerology, Clinical County Emergency Hospital Bihor, 410169 Oradea, Romania; 3 Department of Toxicology, “Victor Babes” University of Medicine and Pharmacy, Eftimie Murgu Square, No. 2, 300041 Timisoara, Romania; andradaiftode@umft.ro (A.I.); diana.haj-ali@umft.ro (D.H.-A.); cristina.grosu@umft.ro (C.D.); cadehelean@umft.ro (C.-A.D.); 4 Research Centre for Pharmaco-Toxicological Evaluation, “Victor Babes” University of Medicine and Pharmacy, Eftimie Murgu Square, No. 2, 300041 Timisoara, Romania; 5 Department of Pathology, Clinical County Emergency Hospital of Arad, Andrenyi Karoly Str., No. 2–4, 310037 Arad, Romania; 6 Department of Pathology, “Pius Brinzeu” Clinical County Emergency Hospital Timisoara, Liviu Rebreanu Boulevard, No. 156, 300723 Timisoara, Romania; 7 Department of Surgical Disciplines, Faculty of Medicine and Pharmacy, University of Oradea, Universității Str., No. 1, 410087 Oradea, Romania; ancahuniadi@gmail.com; 8 Department of Surgery, Pelican Hospital, Corneliu Coposu Str., No. 2, 410450 Oradea, Romania; 9 Department of Surgery I, Faculty of Medicine, “Victor Babes” University of Medicine and Pharmacy, Eftimie Murgu Square, No. 2, 300041 Timisoara, Romania; office@medcom.ro; 10 CBS Medcom Hospital, Popa Sapca Str., No. 12, 300047 Timisoara, Romania; 11 1st Clinic of Obstetrics and Gynecology, “Pius Brinzeu” Clinical County Emergency Hospital Timisoara, Liviu Rebreanu Boulevard, No. 156, 300723 Timisoara, Romania; dr.solomonciprian@protonmail.com; 12 Doctoral School Department, “Victor Babes” University of Medicine and Pharmacy, 300041 Timisoara, Romania; valentin.iovin@umft.ro; 13 Center of Immuno-Physiology and Biotechnologies, Department of Functional Sciences, “Victor Babes” University of Medicine and Pharmacy, 300041 Timisoara, Romania; 14 Department of Functional Sciences, Physiology, Centre of Immuno-Physiology and Biotechnologies (CIFBIOTEH), “Victor Babes” University of Medicine and Pharmacy, 300041 Timisoara, Romania; 15 Faculty of Medicine and Pharmacy, University of Oradea, 1 Decembrie Ave., No. 10, 410073 Oradea, Romania; ovidiu.tica@gmail.com; 16 Preclinical Sciences Department, Faculty of Medicine and Pharmacy, University of Oradea, Universității Str., No. 1, 410087 Oradea, Romania

**Keywords:** pregnancy-associated melanoma, melanoma in situ, dermoscopy, G protein-coupled oestrogen receptor (GPER), melanocortin-1 receptor, onco-fetal immune tolerance, PRAME, p16, dermoscopic surveillance

## Abstract

**Background and Clinical Significance**: Pregnancy-associated melanoma (PAM) is clinically challenging because the physiological pigmentary and naevus changes in pregnancy can obscure early malignancy, and the long-standing assumption that the hormonal milieu of pregnancy drives melanocytic transformation continues to shape clinical expectations. We present a case of postpartum-persistent melanoma in situ and use it to anchor a molecular reappraisal of that hormonal hypothesis. **Case Presentation**: We describe the clinical, dermoscopic, histopathological, and immunohistochemical findings in a 32-year-old woman with a peri-umbilical naevus that changed during the third trimester and persisted twelve months postpartum, and we review the hormonal, immunological, and diagnostic literature relevant to PAM. Dermoscopy showed a multicomponent pattern with asymmetry, irregular structureless areas, peripheral pseudopods, and central regression; excisional biopsy demonstrated an atypical intraepidermal melanocytic proliferation without dermal invasion (melanoma in situ, pTis cN0 cM0, Stage 0), with Melan-A positivity and reduced p16. The reviewed evidence indicates that melanomas lack classical oestrogen/progesterone receptors and that the dominant oestrogen and α-MSH signals act through non-classical, differentiating pathways (GPER, MC1R) that are growth-suppressive rather than oncogenic, while pregnancy provides an immunologically permissive and diagnostically obscuring context. **Conclusions**: Pregnancy is better understood not as a hormonal driver of melanoma but as a permissive and obscuring state in which the principal preventable harm is diagnostic delay; suspicious lesions in pregnant or postpartum women warrant the same urgency as in other patients, and structured dermoscopic surveillance of at-risk women is the rational response.

## 1. Introduction

Pregnancy-associated melanoma (PAM) represents one of the most challenging malignancies diagnosed during the reproductive period, owing to the complex hormonal, immunological, and pigmentary changes occurring throughout pregnancy [1,2]. Melanoma is among the most frequently diagnosed cancers during pregnancy, with an increasing incidence reported in women of childbearing age over recent decades [3,4,5]. Although substantial progress has been achieved in the diagnosis and management of cutaneous melanoma, the relationship between pregnancy and melanoma progression remains incompletely understood and continues to be debated in the current literature [6,7,8,9].

Physiological changes in pregnancy can alter melanocyte biology and skin pigmentation. Increased levels of estrogen, progesterone, α-melanocyte-stimulating hormone, and various growth factors may induce generalized hyperpigmentation and visible changes in pre-existing melanocytic nevi [1,10]. Enlargement, darkening, and dermoscopic changes in nevi during pregnancy have been described in several studies, particularly in areas subject to mechanical stretching such as the abdomen and breasts [2,11]. However, the biological plausibility of direct hormonal stimulation of melanocyte proliferation has been challenged by the finding that human melanocytes express estrogen and progesterone receptors at low levels, suggesting that the observed changes may be only partially mediated by classical steroid receptor pathways [12]. Despite many of these changes being considered physiological and transient, differentiating benign pregnancy-related nevus modifications from early malignant transformation remains a major diagnostic challenge for dermatologists and pathologists [13].

The prognostic significance of PAM remains debated. Earlier studies suggested a possible worse prognosis in pregnant patients with melanoma, raising the question of hormonal stimulation of tumor progression [14,15,16]. Nevertheless, more recent data indicate that pregnancy itself may not worsen melanoma-specific survival independently, provided prompt diagnosis and appropriate therapeutic management are implemented [6,7,17,18,19]. Delayed diagnosis remains a significant problem, as physiological pigmentary changes during pregnancy can lead to underappreciation of concerning melanocytic lesions by both patients and health care providers [4,20,21].

Dermoscopy currently represents an essential non-invasive diagnostic tool in the evaluation of melanocytic lesions, significantly improving the early detection of melanoma while reducing unnecessary surgical excisions [22,23]. Modern dermoscopic analysis enables the identification of subtle malignant structures that may not be clinically evident, including asymmetry, atypical pigment network, irregular globules, regression structures, pseudopods, and multicomponent patterns [24,25]. In pregnancy-associated lesions, however, interpretation may become particularly difficult because certain physiological changes can partially overlap with melanoma-associated dermoscopic features [1,2]. Persistent asymmetry, progressive structural disorganization, marked color variegation, or postpartum persistence of atypical findings should therefore prompt histopathological examination rather than conservative observation [8,26].

Histopathological evaluation continues to be the gold standard for diagnosing melanoma, and immunohistochemical markers, including Melan-A and p16, offer additional diagnostic assistance in difficult melanocytic proliferations [27,28,29]. Recent studies have highlighted the increasing significance of combined immunohistochemical panels—including PRAME alongside p16—to distinguish atypical benign nevi from melanoma, particularly in diagnostically unclear lesions [28,29,30].

In this article, we present the case of a young woman who developed progressive dermoscopic modifications of a pre-existing melanocytic lesion during pregnancy, with persistence of atypical features postpartum, in whom clinicopathological and immunohistochemical correlation ultimately established the diagnosis of melanoma in situ. We then use this case as the anchor for a focused molecular review, reappraising the long-standing hypothesis that the hormonal milieu of pregnancy drives melanocytic transformation and integrating the hormonal, immunological, and diagnostic evidence into a coherent account that motivates dermatoscopic surveillance of at-risk women during pregnancy and the postpartum period.

## 2. Case Presentation

A 32-year-old woman (G1P1) was referred to the Dermatology Department for evaluation of a progressively changing pigmented lesion in the right peri-umbilical abdominal area, 12 months postpartum. The patient was of Fitzpatrick skin phototype II, with approximately 25 melanocytic nevi distributed over the body and no other clinically atypical nevi. She reported a positive history of severe sunburns during childhood. There was no personal or family history of melanoma or other cutaneous malignancies, no chronic medication use, and no relevant comorbidities. Full skin examination revealed no additional suspicious melanocytic lesions, and there was no palpable regional lymphadenopathy or systemic symptoms.

The patient reported that the lesion was a longstanding melanocytic nevus that had been clinically stable for several years before pregnancy. She noted progressive enlargement of the lesion, accentuation of pigmentation, and increased border irregularity during the third trimester of gestation. The lesion retained atypical clinical features and did not regress postpartum, despite term delivery by caesarean section. The patient presented to dermatology approximately 12 months after the onset of the changes.

Dermatologic examination revealed an asymmetrical, round-to-oval pigmented lesion measuring approximately 1.5 × 2 cm in the right peri-umbilical abdominal region. The lesion displayed irregular, poorly defined borders and a heterogeneous, multilobulated appearance with dark-brown to black pigmentation (Figure 1). Areas of color variegation were observed, including dark-brown, reddish-brown, and greyish-white regression-like zones. No satellite pigmentation or regional lymphadenopathy was identified.

Dermoscopy demonstrated marked asymmetry of color and structure, with a multicomponent pattern composed of irregular dark structureless areas, heterogeneous brown-to-black pigmentation, peripheral pseudopod-like projections, and a central pink-whitish regression-like area (Figure 2). No recognizable benign dermoscopic pattern was identified, and the overall architectural disorganization raised strong suspicion for melanoma. Application of the 7-point checklist [24] yielded a high score, further supporting the indication for excisional biopsy.

The patient underwent excisional biopsy with 5 mm safety margins, in accordance with current guidelines for suspicious melanocytic lesions [23,24]. Hematoxylin and eosin staining revealed an atypical melanocytic proliferation confined to the epidermis, composed of irregularly distributed isolated melanocytes and small nests within the superficial epidermal layers (Figure 3). No dermal invasion was identified, allowing classification as melanoma in situ. On review of the excision specimen, no unequivocal residual benign naevus component—dermal naevoid nests or congenital-pattern architecture—was identified at the base of the lesion, although a pre-existing naevus could not be entirely excluded on the available material. The papillary dermis demonstrated pigmented macrophages and a moderate chronic inflammatory infiltrate, findings that correlated with the regression-like structures identified dermoscopically. Based on these findings, the lesion was staged as pTis cN0 cM0 (AJCC 8th edition, Stage 0). Sentinel lymph node biopsy was not indicated, as it is not recommended for melanoma in situ [23,24,31].

Immunohistochemical analysis demonstrated positive Melan-A staining, which highlighted the intraepidermal melanocytic proliferation and confirmed the melanocytic nature of the lesion (Figure 4), together with reduced p16 expression (Figure 5), supporting the interpretation of an atypical, dysregulated melanocytic proliferation in correlation with the histopathological findings.

PRAME (Preferentially Expressed Antigen in Melanoma) immunohistochemistry was not performed in this case; the rationale for regarding the diagnosis as secure despite its absence is addressed in the Discussion Section (Section 3.8).

Following complete surgical excision, the patient was enrolled in a structured dermatologic follow-up program comprising clinical examination and dermoscopic surveillance. At 5 months of follow-up, there was no evidence of local recurrence or new suspicious melanocytic lesions, and the next scheduled review is upcoming. Long-term follow-up is planned, including total body skin examination, dermoscopic surveillance, and patient education on self-examination. The sequence of key clinical events is summarized in Figure 6.

## 3. Discussion

Pregnancy-associated melanoma remains a clinically challenging and highly relevant issue in dermato-oncology, both because melanoma is among the most frequently diagnosed malignancies during the reproductive period and because pregnancy represents a biological and clinical milieu in which pigmentary changes may be difficult to interpret [1,2,3,4,5,9]. Endocrine, immunological, vascular, and mechanical changes during gestation can affect melanocyte activity and alter the clinical and dermoscopic presentation of pre-existing melanocytic nevi [1,6]. Both patients and clinicians may therefore be tempted to attribute nevus growth or darkening to physiological pregnancy-related changes. In the sections that follow, we use the present case to anchor a focused molecular reappraisal of the hormonal hypothesis, before returning to the diagnostic and management lessons it illustrates.

### 3.1. Defining Pregnancy-Associated Melanoma and the Present Case

It is important to note that the present case illustrates a borderline scenario regarding the very definition of PAM. Although classically defined as melanoma diagnosed during pregnancy or within one year postpartum, several authors extend the definition up to two years after delivery, given that pregnancy-related biological effects may persist beyond the immediate postpartum period [4,8,9,15]. Our patient was diagnosed approximately 12 months postpartum, with documented dermoscopic changes originating during the third trimester—placing the case at the upper limit of the most restrictive PAM definition and well within the broader one. This temporal heterogeneity in the literature has direct implications for both epidemiological reporting and clinical surveillance recommendations.

Epidemiologically, melanoma is the malignancy most frequently diagnosed during gestation, and roughly one third of all melanomas in women are diagnosed during the childbearing years [7,22]. In a recent multicentre European series, PAM represented 5.5% of melanomas in women aged 15–49, arose on a pre-existing naevus in more than half of cases, and presented in younger women with higher naevus counts [15]—a profile that matches the present patient. Reported incidence figures of the order of 45 per 100,000 pregnancies, together with the conspicuous pigmentary and naevus changes in gestation, gave rise to a long-standing and intuitively appealing hypothesis: that the hormonal milieu of pregnancy acts as a biological driver of melanocytic transformation and tumour progression [2,3,12,20]. The current evidence increasingly supports the view that pregnancy per se is not necessarily an independent adverse prognostic factor when melanoma is diagnosed early and managed appropriately [6,7,13,20]; the main clinical risk appears to lie less in pregnancy as a direct biological promoter and more in diagnostic delay when suspicious changes are misinterpreted as benign gestational pigmentation. The remainder of this discussion re-examines that hormonal-driver hypothesis at the molecular level and argues that, in its strong form, it is not well supported by the available molecular evidence.

### 3.2. Reappraising the Hormonal-Driver Hypothesis: Classical Hormone Receptors

The hormonal-driver hypothesis presupposes that the steroid surge of pregnancy is transduced within melanocytes by classical nuclear hormone receptors. The available evidence does not support this premise. Immunohistochemical analysis of pregnancy-associated melanomas detected no nuclear oestrogen or progesterone receptor expression [32], and in a larger series of primary melanomas essentially all tumours were oestrogen-receptor-α-negative [33]. Where a classical receptor is expressed in melanocytic lesions it is predominantly oestrogen receptor β, an isoform that behaves as a tumour suppressor: its expression declines with tumour progression, correlates inversely with Breslow thickness, and selective ERβ agonists inhibit melanoma cell growth in vitro [33,34]; a recent review synthesising oestrogen-receptor expression profiles across melanocytic lesions likewise concludes that oestrogen exerts a predominantly inhibitory, rather than mitogenic, effect on melanoma cells [35].

The near-absence of the receptors required for a classical genomic mitogenic response is difficult to reconcile with the notion of pregnancy as a direct hormonal driver of melanoma, and it reframes the central question: how, then, are the undeniable effects of pregnancy on melanocytes actually mediated?

### 3.3. Non-Classical, Differentiating Signalling: GPER and MC1R

The answer increasingly points to non-classical, membrane-initiated signalling—and, crucially, to a signal that is differentiating rather than transforming. Oestrogen exerts its principal effect on melanocytes through the G protein-coupled oestrogen receptor (GPER/GPR30) rather than through nuclear receptors, and this pathway is tumour-suppressive: GPER activation drives a differentiated, pigmented, slowly proliferating phenotype via cAMP–protein kinase A signalling and protein-kinase-A-dependent degradation of c-Myc, and, notably, systemic GPER agonism cooperates with immune-checkpoint blockade to produce durable tumour control in preclinical melanoma models [36]. Consistent with engagement of this axis in vivo, GPER protein is expressed at markedly higher levels in pregnancy-associated than in non-pregnancy-associated melanomas, and GPER-positive tumours display lower Breslow thickness, lower mitotic rate, and denser peritumoural lymphocytic infiltration [33]. A clinical-grade GPER agonist reproduces these growth-inhibitory, differentiating effects in melanoma cells, underscoring the therapeutic—rather than oncogenic—valence of this pathway [37].

The same logic accounts for the pigmentary hallmarks of pregnancy: α-melanocyte-stimulating hormone acts on the melanocortin-1 receptor (MC1R) to raise cAMP and activate the protein kinase A–CREB–MITF cascade that governs melanogenesis, so that gestational hyperpigmentation and naevus darkening reflect activation of the melanocyte differentiation programme, not its transforming one [38,39,40]. Any genuinely pro-tumorigenic contribution of pregnancy, where it exists, appears to operate through non-hormonal routes—most plausibly the placental metalloproteinase PAPP-A, which liberates bioactive insulin-like growth factor-1 and can promote melanoma migration and invasion—although this evidence remains confined to in vitro and xenograft systems [41]. The pregnancy-related hormonal and biological milieu, its effects on melanocytic lesions, and the resulting diagnostic implications are summarized in Figure 7.

At the level of individual signalling cascades, the differentiating valence of these pathways becomes explicit (Figure 8). Both ligand systems converge on the same second messenger: oestrogen, acting through membrane GPER, and α-melanocyte-stimulating hormone, acting through MC1R, each engage Gs-coupled adenylyl cyclase to raise intracellular cAMP and activate protein kinase A (PKA). Downstream, PKA both drives the CREB–MITF axis that induces the melanogenic enzymes tyrosinase, TYRP1 and DCT—the molecular basis of gestational hyperpigmentation—and promotes the degradation of c-Myc, enforcing cell-cycle exit rather than entry [36,38,39,40]. GPER engagement additionally restores major histocompatibility complex class I expression and tumour immunogenicity, which is why pharmacological GPER agonism synergises with PD-1 blockade in preclinical models [36,37]. The single pathway that runs counter to this differentiating tone is non-hormonal: placental PAPP-A, a metalloproteinase that cleaves IGFBP-4 to liberate bioactive IGF-1, can activate IGF-1R–PI3K/AKT signalling and promote melanoma migration and invasion, although this has been demonstrated only in vitro and in xenografts [41].

### 3.4. Molecular Taxonomy and Drivers of the Lesion

A molecular reappraisal of pregnancy-associated melanoma is incomplete without situating the lesion within the genomic taxonomy of melanoma, because that taxonomy determines what actually transforms a melanocyte—and pregnancy figures nowhere among its drivers. In the integrated classification of melanocytic neoplasia, cutaneous melanomas segregate by degree of cumulative sun damage and by initiating mutation: low cumulative-sun-damage (low-CSD) melanomas, which characteristically arise on intermittently exposed trunk and proximal-limb skin and frequently in association with a pre-existing naevus, are typically initiated by BRAF V600E and signal through the MAPK (RAS–RAF–MEK–ERK) cascade, whereas high-CSD and acral or mucosal melanomas follow distinct genetic routes [42,43,44,45]. The present lesion—a long-standing, naevus-associated, peri-umbilical truncal melanoma in a young woman of phototype II with a history of intermittent intense sun exposure—fits the low-CSD, BRAF-pathway profile, the context in which the gestational changes described above played out. Although BRAF V600E predominates in this low-CSD context, a minority of cutaneous melanomas are instead initiated by NRAS or NF1 loss of function, and the in situ lesion characteristically carries a dense ultraviolet-induced mutational signature—itself a fingerprint of the sun-driven, not hormone-driven, origin of the disease [44,45].

The transformation of such a naevus is a stepwise, multi-hit process rather than a single hormonal event (Figure 9). A BRAF- or NRAS-mutant melanocyte first enters oncogene-induced senescence, a protective arrest enforced by the CDKN2A-encoded tumour suppressor p16^INK4a^; escape from this arrest requires additional lesions, canonically a TERT promoter mutation that reactivates telomerase and licenses unlimited replication, followed by loss of CDKN2A function and, ultimately, disruption of PTEN and TP53 at the transition to invasion [42,45,46,47]. This sequence aligns with the present case: the reduced p16 immunoexpression observed in the lesion is the histological footprint of CDKN2A dysregulation acquired late in the in situ phase, while its confinement to the epidermis indicates that the invasion-associated drivers had not yet been engaged. PRAME, the ancillary marker recommended above, is a cancer-testis antigen whose epigenetic de-repression accompanies malignant transformation and whose diffuse expression would have provided orthogonal molecular confirmation of this progression [30,48,49]. None of these events—BRAF mutation, telomerase reactivation, CDKN2A loss—is hormonally driven; the gestational milieu modulates the melanocyte phenotype but does not by itself supply the oncogenic hits.

One genuinely molecular link does, however, connect the pigmentary biology of pregnancy to melanoma risk, and it runs through MC1R (Figure 10). The same receptor whose wild-type signalling produces the protective, eumelanin-rich differentiation programme described above is, when carried in its common loss-of-function “red-hair-colour” (RHC) variants (e.g., R151C, R160W, D294H), a potent risk allele. Attenuated MC1R–cAMP signalling shifts melanin synthesis towards pheomelanin, which not only affords poorer ultraviolet shielding but generates oxidative DNA damage through an ultraviolet-radiation-independent mechanism [50]; in parallel, intact MC1R signalling normally enhances nucleotide-excision repair via PKA-dependent phosphorylation of ATR and recruitment of XPA, and stabilises PTEN, so that loss-of-function variants simultaneously impair genome maintenance and de-repress PI3K/AKT signalling [51,52,53]. The molecular consequence is a higher somatic mutation burden and melanoma risk in variant carriers [54]—precisely why MC1R-variant and red-haired women, already identified clinically as a surveillance priority, have a mechanistic claim to that priority. The principal molecular determinants discussed here, the direction of their effect, and the strength of the supporting evidence are summarised in Table 1.

### 3.5. An Immunologically Permissive Microenvironment

If pregnancy does not drive melanoma hormonally, two other features of gestation are nonetheless germane to the disease; the first is immunological. Maternal tolerance of the semi-allogeneic fetus is sustained by regulatory T cells, HLA-G, indoleamine 2,3-dioxygenase, and the PD-1/PD-L1 axis—precisely the pathways that tumours co-opt to evade immune destruction—and recent work has formalised this overlap, identifying progesterone-driven, onco-fetal tolerance checkpoints shared between the maternal–fetal interface and the tumour microenvironment (Figure 11) [55,56,57,58,59]. The clinical correlate within PAM is telling: pregnancy-associated tumours contain significantly fewer tumour-infiltrating lymphocytes than melanomas in non-pregnant women [15], pointing to a less immunogenic, more permissive microenvironment. A pregnancy-enhanced lymphangiogenic drive has additionally been demonstrated in experimental melanoma and may facilitate dissemination—a mechanistic counterpoint that should be acknowledged even though it is not borne out in modern clinical outcomes [60].

This molecular overlap is mirrored at the cellular level. At the maternal–fetal interface, decidual natural killer cells are held in a hypofunctional, non-cytotoxic state through HLA-G engagement of their inhibitory KIR and LILRB receptors, tolerogenic macrophages and dendritic cells predominate, and a FoxP3^+^ regulatory T-cell-rich, type-2-skewed compartment is established [58]. The pregnancy-associated tumour appears to recapitulate precisely this “cold”, immune-excluded architecture, offering a unifying account of its lymphocyte-poor microenvironment. The hormonal and immune axes are, moreover, not fully separable: oestrogen acting through ERα in tumour-associated macrophages can itself polarise them toward an immunosuppressive, M2-like state that promotes CD8^+^ T-cell exhaustion and underlies part of the sex difference in melanoma immunotherapy response [61]. The net effect of the gestational milieu on tumour immunity is therefore best read as a balance of differentiating and tolerising signals rather than a single, uniform direction.

### 3.6. A Diagnostically Obscuring Milieu: Physiological Versus Suspicious Naevus Change

The second feature is diagnostic. The benign naevus changes in pregnancy are real but stereotyped: symmetrical enlargement and pigmentary accentuation concentrated on the mechanically stretched abdomen and back, without change in dermoscopic architecture and without the appearance of new lesions [1,11,20,25,62,63]. These changes are typically diffuse and not associated with major disruption of the global dermoscopic pattern, and they tend to stabilize or regress postpartum. Such physiological changes obscure the recognition of genuinely atypical lesions, blurring the boundary between physiological adaptation and early malignancy; persistence or progression of atypical features after delivery should therefore be regarded as a warning sign rather than a normal continuation of gestational change.

The main clinical and dermoscopic differences between physiological pregnancy-related nevus changes and suspicious melanoma-associated changes are summarized in Table 2.

In the present case, the clinical and dermoscopic features clearly exceeded the spectrum of physiological pregnancy-associated nevus modification. Twelve months postpartum, the lesion remained clinically atypical, with progressive enlargement, darker pigmentation, irregular borders, and marked asymmetry, while dermoscopy revealed a multicomponent pattern with irregular dark structureless areas, heterogeneous brown-black pigmentation, peripheral pseudopod-like projections, and a central pink-whitish regression-like area. The most important diagnostic clue was not a single isolated feature but rather the convergence of several suspicious elements: clinical evolution, postpartum persistence, dermoscopic asymmetry, color variegation, architectural disorganization, and regression. In melanocytic lesions, the overall pattern often carries greater diagnostic significance than any individual structure; a benign nevus may darken or enlarge slightly during pregnancy if the global architecture is preserved, whereas histopathological confirmation should be obtained when the lesion loses its symmetry, displays multiple colors, develops regression structures, and fails to regress after delivery.

### 3.7. Prognosis Reconsidered

These two features—an immunologically permissive milieu and a diagnostically obscuring one—together help to resolve the long-running controversy over PAM prognosis. Earlier meta-analyses reported worse outcomes, with pooled estimates of increased melanoma-specific mortality and recurrence in pregnancy-associated disease [16,64]. More recent and methodologically stronger analyses, however, find no independent adverse effect once stage is accounted for: a 2025 meta-analysis of more than twenty-nine thousand patients reported overall survival that, if anything, favoured pregnant women, while contemporary registry and cohort data show no significant difference in recurrence-free or melanoma-specific survival [6,7,13,15]. The most parsimonious reading is that any residual excess in tumour thickness or stage at presentation reflects delayed detection rather than an intrinsically more aggressive biology—an interpretation that aligns precisely with the molecular picture of a non-oncogenic, even differentiating, hormonal signal operating within a permissive and obscuring context.

### 3.8. Clinicopathological and Immunohistochemical Correlation in the Present Case

Dermoscopic regression structures merit particular attention. Histopathologically, areas of gray-white or gray-blue coloration may correspond to fibrosis, melanophages, inflammation, and partial regression of the melanocytic tumour components. In our case, the dermoscopic regression areas correlated with pigmented macrophages and chronic inflammatory infiltrate in the papillary dermis, supporting strong clinicopathological concordance and demonstrating that the dermoscopic abnormalities reflected true histological alterations of the lesion rather than pigmentary changes related to pregnancy alone [65].

Histopathological examination was decisive, demonstrating an atypical melanocytic proliferation confined to the epidermis without evidence of dermal invasion. This finding established the diagnosis of melanoma in situ and allowed appropriate staging as pTis cN0 cM0 (Stage 0). The diagnosis was further supported by immunohistochemistry: Melan-A highlighted the intraepidermal melanocytic component, while reduced p16 expression supported the interpretation of a dysregulated atypical melanocytic lesion in the appropriate morphological context. These markers should not be interpreted in isolation; immunohistochemistry is most useful in melanocytic pathology when applied in the context of clinical evolution, dermoscopic criteria, and conventional histopathological morphology. In this case, the combination of postpartum persistence, dermoscopic architectural disorder, intraepidermal atypical melanocytic proliferation, Melan-A positivity, and reduced p16 expression supported the final diagnosis of melanoma in situ. The diagnostic value of p16-based immunohistochemistry as an adjunct in the assessment of atypical melanocytic and epithelial proliferations is well recognised in other settings as well [66].

A point relevant to the interpretation of this case is the relationship between the reported clinical history and the histopathological findings. No unequivocal residual benign naevus component could be identified at the base of the intraepidermal atypical proliferation, and a pre-existing naevus could not be confidently excluded on the available material; the case is therefore compatible with two interpretations. The first is that a pre-existing melanoma in situ remained clinically indolent for several years and became clinically and dermoscopically manifest during the third trimester and postpartum period—a dormant in situ lesion unmasked, rather than initiated, by the immunologically permissive milieu of gestation described in Section 3.5. The second is that malignant transformation arose within a pre-existing naevus during the third trimester itself, a scenario more compatible with a direct biological contribution of pregnancy to melanocytic transformation. The present material does not allow these possibilities to be distinguished with certainty; the molecular argument developed below is accordingly framed as one of biological plausibility rather than proof, the temporal association between gestation and the clinical emergence of the lesion being compatible with either an unmasking or a transformation mechanism.

A limitation of the present case is the lack of PRAME (Preferentially Expressed Antigen in Melanoma) immunohistochemistry, which has emerged in recent years as a valuable adjunct marker in the differential diagnosis of melanocytic neoplasms, particularly when combined with p16 [30,48,49,67]. Diffuse PRAME positivity favors a malignant interpretation, and combined PRAME/p16 panels have been shown to improve diagnostic accuracy in atypical or borderline melanocytic proliferations. In our case, the diagnosis of melanoma in situ was firmly established on the basis of clinical, dermoscopic, histopathological, and Melan-A/p16 immunohistochemical findings; nevertheless, the addition of PRAME would have provided further diagnostic reinforcement and is recommended in similar future cases when available.

### 3.9. Management, Surveillance, and Limitations

From a management perspective, this case reinforces a key principle: suspicious melanocytic lesions should not be observed passively during pregnancy when melanoma is suspected clinically or dermoscopically. Current recommendations favor prompt excisional biopsy under local anesthesia when indicated, since diagnostic delay carries greater risk than the procedure itself [2,8,23,24,31]. Such preventable delay is often related to the misconception that dermatologic surgery should be postponed until after delivery; in reality, early biopsy and adequate excision are paramount for maternal safety and can usually be performed without compromising fetal well-being [9,68].

A further important aspect of this case is the postpartum interval. The patient presented approximately 12 months after the onset of the changes with a clinically atypical and dermoscopically suspicious lesion. This delay highlights a recurrent problem in PAM, in which patients may normalize pigmentary changes during pregnancy and clinicians may downplay lesions in the absence of systemic symptoms or a personal history of melanoma [69]. The practical corollary of the molecular picture is that the principal preventable harm in PAM is diagnostic delay, and the rational response is structured surveillance of the women most at risk: those with high naevus counts or the dysplastic-naevus syndrome, with CDKN2A alterations or familial melanoma, with loss-of-function MC1R variants and the red-hair phenotype, or with a personal history of melanoma. Baseline and sequential dermoscopic and digital monitoring through gestation and the postpartum period—with prompt biopsy of any architecturally atypical or postpartum-persistent lesion and judicious use of immunohistochemical adjuncts such as PRAME and p16—offers a means of removing that delay without medicalising the normal pigmentary changes in pregnancy [20,23,24,30,48]. Education on self-examination and early dermatologic evaluation during pregnancy should therefore be considered part of preventive care, particularly for women with multiple nevi, atypical nevi, or changing lesions [9,20,70]. Looking further ahead, molecular tools are beginning to complement dermoscopic surveillance: PRAME transcript detection and tumour-informed circulating tumour DNA assays can flag residual or recurrent disease before it becomes clinically or radiologically apparent, although their performance in the in situ setting and during pregnancy remains to be defined [71].

Fortunately, the prognosis in this case is excellent due to diagnosis at the melanoma in situ stage and complete surgical excision. Long-term dermatologic follow-up remains essential and includes total body skin examinations, dermoscopic surveillance, and patient education on the ABCDE criteria and self-examination. The main long-term risk is not recurrence of the completely excised lesion but rather the development of additional primary melanomas, since a previous melanoma—even in situ—increases the need for structured follow-up [23,24,31]. The principal limitation of this report is inherent to its single-case design: while the case illustrates the molecular argument compellingly, it cannot by itself establish causal or prognostic claims, which rest on the cohort and mechanistic literature reviewed above.

Taken together, the hormonal, genomic, immunological and diagnostic threads converge on a single, clinically actionable synthesis (Figure 12).

## 4. Conclusions

Melanocytic lesions in pregnancy should not be automatically attributed to physiological gestational changes. Pregnancy may cause darkening or slight enlargement of pre-existing nevi; however, such modifications should remain proportionate, symmetrical, and dermoscopically organized. When a lesion develops progressive asymmetry, architectural disorder, multiple colors, regression structures, pseudopod-like projections, or persistence after delivery, a low threshold for biopsy is warranted.

This case demonstrates that melanoma in situ may clinically mimic pregnancy-related nevus modification, especially when dermatologic consultation is delayed, and that postpartum persistence should not be considered benign in the presence of dermoscopic disorganization. Early excision allowed diagnosis at the in situ stage, before progression to invasive melanoma.

At the molecular level, the evidence reframes pregnancy not as a hormonal driver of melanocytic transformation but as an immunologically permissive and diagnostically obscuring state, in which the dominant hormonal signalling is, if anything, differentiating and protective. The fundamental clinical principle follows directly: suspicious melanocytic lesions in pregnant or postpartum patients should be evaluated with the same urgency and diagnostic rigor as in non-pregnant patients, and at-risk women benefit from structured dermoscopic surveillance through gestation and the postpartum period. Although pregnancy may modify the skin, it should never delay the diagnosis of melanoma.

## Figures and Tables

**Figure 1:**
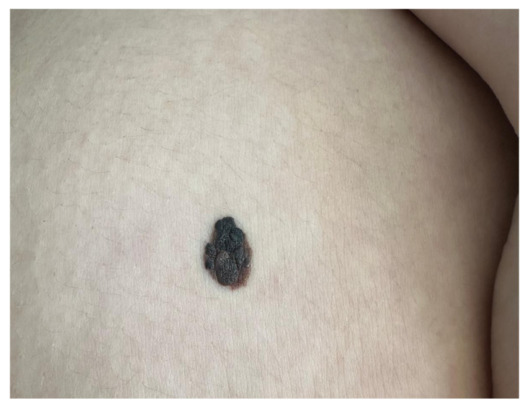
Clinical presentation of the lesion. Asymmetric round-to-oval pigmented lesion located in the right peri-umbilical abdominal area, showing irregular borders, heterogeneous brown-black pigmentation, and marked color variegation.

**Figure 2:**
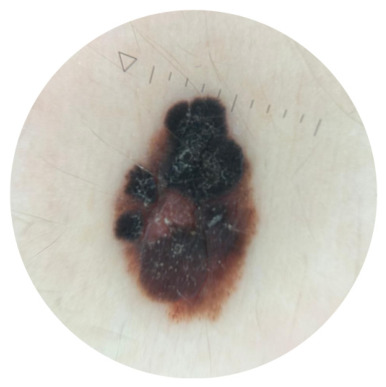
Dermoscopic appearance of the lesion (magnification 5×). Dermoscopy revealed marked asymmetry of color and structure, with a multicomponent pattern composed of irregular dark structureless areas, heterogeneous brown-black pigmentation, peripheral pseudopod-like projections, and a central pink-whitish regression-like area. The absence of a specific benign pattern and the overall architectural disorganization raised strong suspicion for melanoma.

**Figure 3:**
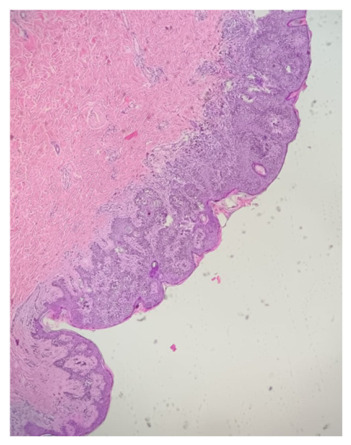
Histopathological examination, hematoxylin and eosin staining, low-power view (magnification 4×). The section shows an atypical intraepidermal melanocytic proliferation arranged as isolated melanocytes and small nests, confined to the epidermis and consistent with melanoma in situ. Pigmented macrophages and a moderate chronic inflammatory infiltrate are visible within the papillary dermis, supporting clinicopathological correlation with the dermoscopic regression-like areas.

**Figure 4:**
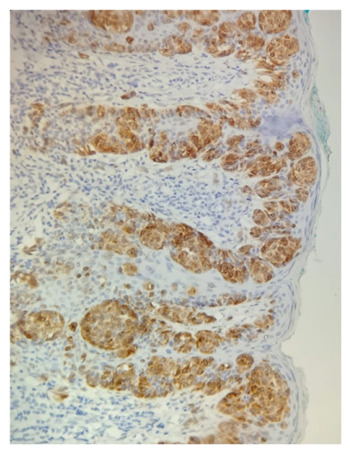
Melan-A immunohistochemistry (magnification 10×). Positive Melan-A staining highlights the atypical intraepidermal melanocytic proliferation, supporting the melanocytic origin of the lesion in correlation with the histopathological findings.

**Figure 5:**
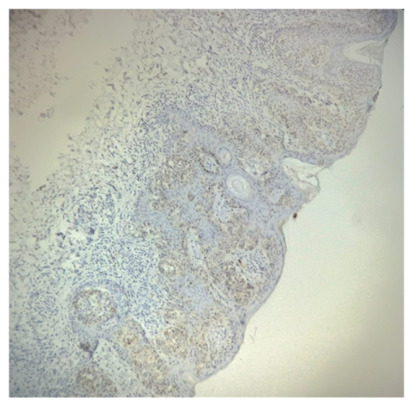
p16 immunohistochemistry (magnification 4×). Reduced p16 expression is observed within the atypical melanocytic proliferation, supporting the interpretation of a dysregulated melanocytic lesion in correlation with the histopathological and dermoscopic findings.

**Figure 6:**
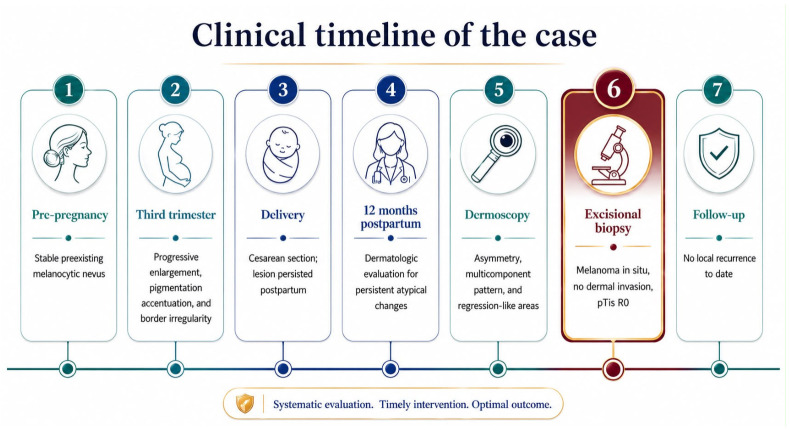
Clinical timeline of the case. Schematic representation of key clinical events, from the pre-pregnancy stable nevus, through gestational dermoscopic changes and postpartum persistence, to excisional biopsy, histopathological diagnosis of melanoma in situ, and subsequent dermatologic follow-up.

**Figure 7:**
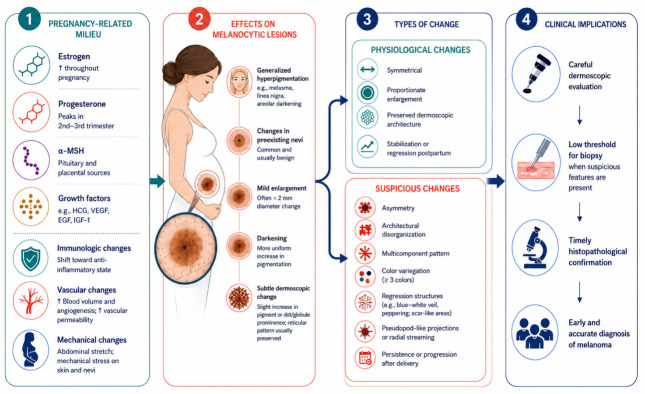
Hormonal influence and diagnostic implications in pregnancy-associated melanocytic lesions. Schematic overview of the pregnancy-related hormonal and biological milieu, its effects on melanocyte activity and melanocytic nevi, the distinction between physiological and suspicious lesion changes, and the main clinical implications for dermoscopic evaluation and timely diagnosis.

**Figure 8:**
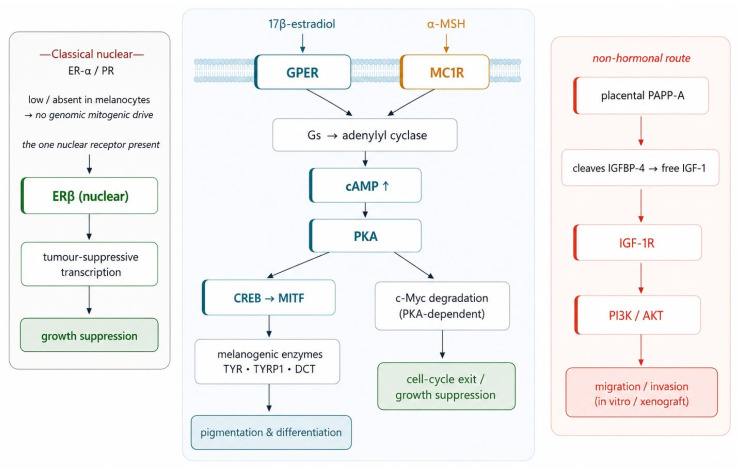
Molecular signalling in the pregnant melanocyte. Oestrogen (via GPER) and α-melanocyte-stimulating hormone (via MC1R) converge on Gs–cAMP–protein kinase A signalling, driving the CREB–MITF melanogenic programme (pigmentation and differentiation) and PKA-dependent c-Myc degradation (growth suppression). The classical receptors ER-α and PR are essentially absent, while ERβ is tumour-suppressive; the only pro-invasive route shown is non-hormonal (placental PAPP-A–IGF-1–PI3K/AKT), supported by in vitro and xenograft data only. See Section 3.3 for detail.

**Figure 9:**
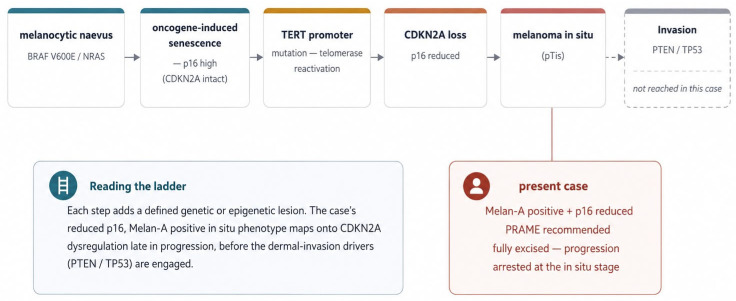
Canonical molecular progression of melanoma (low cumulative-sun-damage, BRAF pathway), from a BRAF/NRAS-mutant naevus through oncogene-induced senescence, TERT-promoter-driven escape and CDKN2A loss to invasion-associated PTEN/TP53 disruption. The present case—Melan-A positive with reduced p16—maps onto CDKN2A dysregulation at the in situ stage. See Section 3.4 for the full sequence.

**Figure 10:**
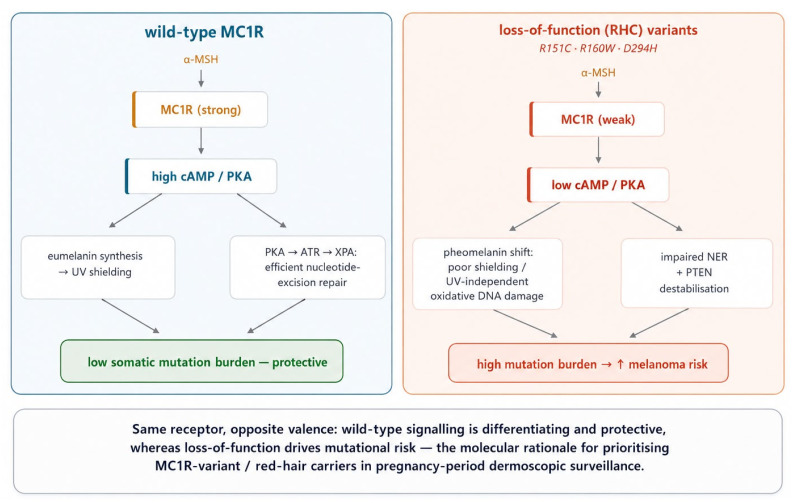
MC1R signalling as a determinant of melanoma risk. Wild-type MC1R–cAMP signalling promotes eumelanin synthesis and efficient nucleotide-excision repair, yielding a low somatic mutation burden; common loss-of-function red-hair-colour variants (e.g., R151C, R160W, D294H) attenuate cAMP signalling, shift synthesis towards pheomelanin with ultraviolet-independent oxidative DNA damage, impair DNA repair and destabilise PTEN, raising the mutation burden and melanoma risk. See Section 3.4 for detail.

**Figure 11:**
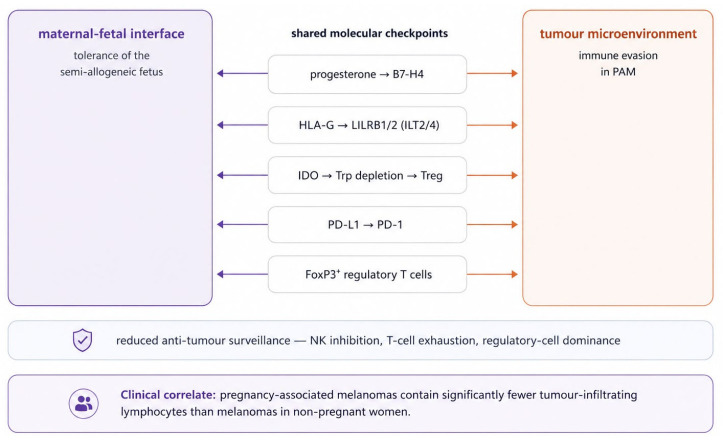
Shared onco-fetal immune-tolerance checkpoints—progesterone-driven B7-H4, HLA-G–LILRB1/2 (ILT2/4) signalling, IDO-mediated tryptophan depletion, the PD-L1/PD-1 axis, and FoxP3^+^ regulatory T cells—deployed both at the maternal–fetal interface and within the tumour microenvironment. See Section 3.5 for detail.

**Figure 12:**
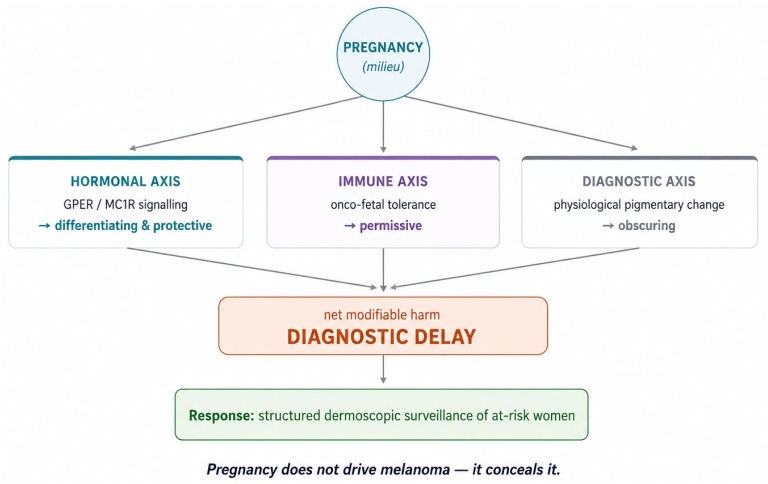
Integrated model of pregnancy and melanoma. Across three axes—hormonal (GPER/MC1R signalling: differentiating and protective), immune (onco-fetal tolerance: permissive) and diagnostic (physiological pigmentary change: obscuring)—the principal modifiable harm that emerges is diagnostic delay, for which structured dermoscopic surveillance of at-risk women is the rational response. The figure also serves as a graphical abstract.

**Table 1:** Molecular determinants in pregnancy-associated melanoma: pathway or mediator, direction of effect, and level of supporting evidence. Direction is given relative to melanocytic transformation; “evidence” indicates the strongest study type currently available.

Pathway/Mediator	Direction of Effect	Evidence	Ref.
Classical ER-α/PR (nuclear)	Absent in melanocytes; no mitogenic drive	Human tumour IHC	[32,33]
ERβ (nuclear)	Tumour-suppressive; declines with progression	Human tissue + in vitro	[33,34]
GPER (membrane oestrogen receptor)	Growth-suppressive, differentiating; ↑ immunogenicity	In vitro + mouse + human	[33,36,37]
MC1R, wild-type (α-MSH/cAMP/PKA)	Differentiation (eumelanin); enhances NER	In vitro + mouse	[38,39,52,53]
MC1R, RHC loss-of-function variants	↑ mutation burden; ↑ melanoma risk	Mouse + human genetic	[50,51]
cAMP/PKA/CREB/MITF	Pigmentation, differentiation (not transformation)	In vitro + mouse	[38,39,40]
Placental PAPP-A → IGF-1 → PI3K/AKT	Pro-migratory/invasive	In vitro/xenograft only	[41]
Onco-fetal checkpoints (B7-H4, HLA-G, IDO, PD-L1, Treg)	Immunologically permissive (evasion)	Human + mechanistic	[15,55,56,57,58]
BRAF/NRAS → MAPK	Initiating oncogenic driver (non-hormonal)	Human genomic	[42,43,44,45]
TERT promoter mutation	Telomerase reactivation/immortalisation	Human genomic	[46,47]
CDKN2A loss (p16)	Senescence escape/progression	Human genomic + case IHC	[42,46]

Note: ↑= increase.

**Table 2:** Physiological versus suspicious melanocytic lesion changes during pregnancy. The table summarizes the main clinical and dermoscopic features that help distinguish benign pregnancy-related nevus modifications from changes that should raise suspicion for melanoma.

Physiological Pregnancy-Related Changes	Suspicious Melanoma-Associated Changes
Mild, symmetrical enlargement	Progressive asymmetrical enlargement
Uniform darkening or pigmentation increase	Marked colour variegation/multiple colours
Preserved global dermoscopic architecture	Architectural disorganization
Regular reticular or globular pattern	Atypical pigment network or multicomponent pattern
Slight increase in dots or globules with symmetrical distribution	Irregular globules or irregular dark structureless areas
Absence of regression structures	Gray-blue, white, or scar-like regression structures
No pseudopods or radial streaming	Pseudopod-like projections or radial streaming
Stabilization or regression after delivery	Persistence or progression postpartum
Changes mainly in mechanically stretched areas	Suspicious changes regardless of anatomical stretching
Conservative monitoring may be appropriate if architecture remains benign	Excisional biopsy is indicated when melanoma is suspected

## Data Availability

The data presented in this study are contained within the article. Further enquiries can be directed to the corresponding author.
